# Usability, reliability, and validity of the Arabic version of the ASK-20 questionnaire in Arab adults

**DOI:** 10.3389/fmed.2025.1595308

**Published:** 2025-05-27

**Authors:** Mohammed S. Alharthi

**Affiliations:** Department of Clinical Pharmacy, College of Pharmacy, Taif University, Taif, Saudi Arabia

**Keywords:** medication adherence, ASK-20 questionnaire, Arabic validation, adherence barriers, reliability, cultural adaptation

## Abstract

**Background:**

Medication adherence is crucial for optimal therapeutic outcomes, yet it remains a significant challenge globally, including in Arabic-speaking populations. Existing tools like the “Adherence Starts with Knowledge 20” (ASK-20) questionnaire assess adherence barriers but lack a validated Arabic version. To address this gap, this study evaluated the usability, reliability, and validity of the Arabic ASK-20 questionnaire among Arab adults.

**Methods:**

A cross-sectional study was conducted with 130 Arabic-speaking adults aged 18 years or older. The ASK-20 questionnaire was translated and culturally adapted using a rigorous linguistic validation process, including forward translation, expert review, and back-translation. Data were collected from November to December 2024. Internal consistency was assessed using Cronbach’s alpha, while floor and ceiling effects evaluated response biases. Statistical analyses included descriptive and inferential statistics, with significance set at *p* < 0.05.

**Results:**

The study sample comprised 69.2% males and 30.8% females, with a mean age of 36.6 years. The Arabic ASK-20 demonstrated acceptable internal consistency (Cronbach’s alpha = 0.751). Key adherence barriers identified included cost-related issues (76.9% ceiling effect) and medication availability (74.6% ceiling effect). The ASK-20 and Total Barrier Count (TBC) scores effectively differentiated between adherence levels, with lower scores observed in the “Good-1” adherence group compared to the “Poor-1” group.

**Conclusion:**

The Arabic ASK-20 is a reliable and valid tool for identifying medication adherence barriers in Arab adults. By systematically assessing barriers such as cost, availability, and knowledge gaps, the tool equips healthcare providers to design targeted, patient-centered interventions. These findings highlight the potential of the Arabic ASK-20 to improve chronic disease management, enhance patient outcomes, and inform public health strategies within Arabic-speaking communities.

## Introduction

Medication adherence is a cornerstone of effective healthcare management, directly impacting therapeutic outcomes, patient safety, and quality of life (1). Non-adherence to prescribed treatments remains a global challenge, even in developed countries, where nearly 50% of patients with chronic illnesses fail to follow their prescribed regimens (1). The economic impact of this issue is substantial, with annual costs per person ranging from $949 to $44,190 due to increased utilization of inpatient, outpatient, and emergency care services (2). These figures emphasize the urgent need to address non-adherence to mitigate avoidable healthcare costs and optimize resource allocation (2). Non-adherence arises from multiple factors, including the complexity of treatment regimens, socioeconomic barriers, and patient-related challenges such as forgetfulness, lack of understanding, or misconceptions about medications (1). For instance, higher medication costs and regimens requiring multiple daily doses are strongly associated with lower adherence rates (3, 4). This issue is particularly alarming in the context of chronic diseases such as diabetes and cardiovascular disorders, where poor adherence exacerbates the risk of disease progression, hospitalization, and adverse health outcomes (5). Addressing adherence challenges requires reliable, validated tools to identify barriers and inform targeted interventions systematically. Evidence suggests that tailored strategies can improve adherence by 20–30%, resulting in better disease management and reduced healthcare costs (6). Among the existing tools, the “Adherence Starts with Knowledge 20” (ASK-20) questionnaire, developed by GlaxoSmithKline in 2008, stands out for its comprehensive evaluation of factors influencing adherence. The ASK-20 consists of 20 items that assess barriers related to patient knowledge, behaviors, and systemic factors, making it applicable across various medical conditions, including asthma, depression, diabetes, and heart failure (7). The development and initial validation of the ASK-20 involved psychometric testing with 605 patients, demonstrating strong reliability (Cronbach’s alpha = 0.85) and the ability to identify clinically actionable adherence barriers (8). Subsequent research confirmed its robust internal consistency (Cronbach’s alpha = 0.76) and ability to differentiate adherence levels using self-reported and objective measures (9). Its cross-cultural applicability has been supported by studies in Japan, where validation among bronchial asthma patients demonstrated similar reliability (Cronbach’s alpha = 0.76) and highlighted its usability in diverse healthcare settings (10). These findings reinforce ASK-20’s versatility in identifying adherence challenges and informing actionable interventions. Despite its global utility, the ASK-20 questionnaire has not been validated in Arabic, limiting its application in Arabic-speaking communities. Chronic diseases such as diabetes, hypertension, and cardiovascular disorders are highly prevalent in the Arab world, where non-adherence to treatment regimens compounds the public health burden. The Middle East and North Africa region has the highest global prevalence of diabetes among adults, with rates projected to rise significantly (11). In Saudi Arabia, hypertension affects 22.66% of adults, while in Egypt, nearly 29.2% of adults aged 30–79 are hypertensive (12, 13). These conditions underscore the urgent need for culturally adapted tools to assess adherence behaviors and barriers in this region. This study addresses this gap by developing and validating an Arabic version of the ASK-20 questionnaire. Through rigorous linguistic and cultural adaptation, the Arabic ASK-20 seeks to provide a reliable tool for evaluating adherence barriers. Specifically, the study aims to assess the usability, reliability, and validity of the Arabic ASK-20 among Arab adults, enabling healthcare providers to design evidence-based interventions tailored to this population.

## Materials and methods

### Study design

This cross-sectional study evaluated the usability, reliability, and validity of the Arabic version of the Adherence Starts with Knowledge 20 (ASK-20) questionnaire among Arab adults. The study was conducted in a single session without follow-up and employed a non-disease-specific approach to ensure applicability across diverse populations.

### Translation and adaptation

The ASK-20 questionnaire was translated into Arabic following a rigorous linguistic validation process. The forward translation was performed by an experienced bilingual linguist (AA), reviewed by an expert panel for cultural relevance, and back-translated into English by an independent translator to confirm semantic equivalence with the original version. The expert review panel, consisting of three bilingual clinical pharmacy and public health experts, assessed the translated version for clarity, cultural appropriateness, and semantic equivalence. Based on their feedback, minor adjustments were made to improve the phrasing of items related to emotional wellbeing and interactions with healthcare providers. The translated questionnaire was then pilot-tested with native Arabic speakers (*n* = 10) to ensure clarity and cultural appropriateness. The original English version of the ASK-20 questionnaire was obtained under a license agreement from Mapi Research Trust (Special Terms No. 102926) (7). The Arabic version was translated and culturally adapted following the methodology and requirements set forth by Mapi Research Trust, including linguistic validation in compliance with ISPOR guidelines (14).

### Participants and recruitment

Participants were recruited using convenience sampling through digital outreach via community social media platforms. The sample size was determined according to established guidelines for questionnaire validation, which recommend a participant-to-item ratio of at least 5:1 to 10:1 for robust psychometric analysis (15). Given that the ASK-20 questionnaire contains 20 items, a minimum sample size between 100 and 200 was considered appropriate. A total of 130 participants were recruited to meet this requirement, allowing for adequate evaluation of internal consistency and response patterns. Invitations and study details were shared in relevant groups and forums, targeting adults fluent in Arabic. Eligible participants included individuals aged 18 years or older who provided informed consent. Exclusion criteria included individuals with cognitive or physical impairments that could hinder their ability to complete the questionnaire.

### Data collection

The data was collected from November 2024 to December 2024. The Arabic version of the ASK-20 questionnaire identified potential barriers to medication adherence. This self-administered tool consists of 20 items rated on a 5-point Likert scale, addressing domains such as forgetfulness, access to medication, cost-related issues, and understanding healthcare provider instructions. Participants received instructions to base responses on general experiences rather than specific diseases or conditions. Demographic information, including age and gender, was also collected.

### Instrument reliability and validation

The reliability of the Arabic version of the ASK-20 questionnaire was assessed using Cronbach’s alpha to evaluate internal consistency, with a value of 0.7 or higher considered acceptable. Floor and ceiling effects were examined by analyzing the distribution of responses for each item, with items showing ≥ 50% responses at the lowest or highest ends flagged for potential bias.

### Statistical analysis

Data analysis relied on SPSS version 27. Descriptive statistics summarized the demographic characteristics of participants, including age and gender, and response patterns for each of the 20 items on the questionnaire. Internal consistency reliability was determined using Cronbach’s alpha, ensuring adequate correlation among questionnaire items. Floor and ceiling effects were assessed by examining the distribution of responses across the 5-point Likert scale. Items with ≥ 50% responses at the lowest or highest extremes were flagged for potential bias. Descriptive statistics compared adherence-related measures across adherence groups, including the ASK-20 total scores and Total Barrier Count (TBC). Means and standard deviations described differences in adherence behaviors. Statistical significance for all analyses adhered to a threshold of *p* < 0.05.

### Ethical approval

Ethical approval for this study was granted by Taif University’s Committee (application number 46 - 068). Key ethical considerations included ensuring confidentiality and obtaining informed consent. Before consenting, participants were informed about the study’s purpose, procedures, potential risks, and benefits. Participation was entirely voluntary, with no coercion involved. Furthermore, the researchers prioritized protecting participants’ privacy and safeguarding their identities and sensitive information.

## Results

The study participants were predominantly male, representing 69.2% of the sample, while females accounted for 30.8%, as shown in Table 1. The age distribution revealed that nearly half of the participants, 49.2%, were in the 18–24 age group, indicating a predominance of younger individuals. The following largest groups included those aged 25–34 years (22.3%), followed by 35–44 years (16.9%), and those above 45 years (11.6%). The mean age of participants was 36.6 years with a standard deviation of 10.05, and ages ranged from a minimum of 29.5 years to a maximum of 60 years. This demographic composition highlights a diverse age range, with a notable concentration of younger participants, reflecting the target population’s characteristics.

**TABLE 1:** Demographic characteristics of study participants.

Characteristic	Count	Percentage (%)
**Age (years)**		
18–24	64	49.2
25–34	29	22.3
35–44	22	16.9
Above 45	15	11.6
**Gender**		
Male	90	69.2
Female	40	30.8

### Internal consistency reliability

The study’s internal consistency reliability was assessed using Cronbach’s Alpha, a measure commonly used to evaluate scale reliability. The calculated Cronbach’s Alpha value was 0.751, indicating acceptable internal consistency for the scale. Additionally, Cronbach’s Alpha based on standardized items was 0.749, supporting the scale’s reliability with its 20 items. These values demonstrate that the items within the scale are sufficiently correlated to measure the intended construct consistently.

### Bias analysis of ASK-20 responses

Table 2 shows that the responses to the ASK-20 questions reveal notable floor and ceiling effects, highlighting areas of potential bias in participants’ medication adherence behaviors. Questions related to forgetting medicines (Questions 1 and 5) displayed modest floor (14.6%) and ceiling effects (11.5%), indicating variability in adherence due to forgetfulness. High ceiling effects were observed in questions addressing skipping or stopping medication due to cost (Question 19: 76.9%) and not having medicine available when needed (Question 20: 74.6%), suggesting these are significant barriers to medication adherence. Additionally, a considerable ceiling effect was noted for participants feeling confident in their medications’ efficacy (Question 7: 25.4%) and understanding their healthcare provider’s instructions (Question 10: 36.2%), reflecting positive adherence. Conversely, inconvenience in taking medications multiple times a day (Question 13) showed the highest floor effect (37.7%), indicating this is less of a concern for most participants. Overall, the findings emphasize the impact of cost, availability, and perceived effectiveness on adherence behaviors.

**TABLE 2:** Descriptive and bias analysis of responses to the Arabic ASK-20 questionnaire.

Question*		Mean (SD)	Median	Floor *n* (%)	Ceiling *n* (%)
1	I just forget to take my medicines sometimes.	2.63 (1.23)	2.0	19 (14.6)	15 (11.5)
2	I run out of my medicine because I don’t get refills on time.	3.11 (1.29)	3.0	12 (9.2)	26 (20.0)
3	My use of alcohol gets in the way of taking my medicines.	3.78 (1.47)	5.0	18 (13.8)	66 (50.8)
4	I worry about how medicine will affect my sexual health.	3.03 (1.31)	3.0	16 (12.3)	26 (20.0)
5	I sometimes forget things that are important to me.	2.63 (1.22)	2.0	19 (14.6)	15 (11.5)
6	I have felt sad, down, or blue during the past month.	2.72 (1.43)	2.0	33 (25.4)	21 (16.2)
7	I feel confident that each one of my medicines will help me.	3.93 (0.86)	4.0	0 (0.0)	33 (25.4)
8	I know if I am reaching my health goals.	3.82 (0.81)	4.0	1 (0.8)	23 (17.7)
9	I have someone whom I can call with questions about my medicines.	4.19 (0.62)	4.0	0 (0.0)	40 (30.8)
10	I understand my doctor’s/nurse’s instructions about take.	4.20 (0.80)	4.0	2 (1.5)	47 (36.2)
11	My doctor/nurse and I work together to make decisions.	3.80 (1.05)	4.0	5 (3.8)	34 (26.2)
12	I need to read and understand pill bottle labels.	4.22 (0.84)	4.0	2 (1.5)	51 (39.2)
13	Taking medicines more than once a day is inconvenient.	1.91 (0.92)	2.0	49 (37.7)	1 (0.8)
14	I have to take too many medicines a day.	3.67 (1.24)	4.0	12 (9.2)	38 (29.2)
15	It is hard for me to swallow the pills I have to take.	3.55 (1.23)	4.0	8 (6.2)	36 (27.7)
16	Taken a medicine more or less often than prescribed?	4.38 (1.16)	5.0	7 (5.4)	92 (70.8)
17	Skipped or stopped taking a medicine because you didn’t think it was working?	4.16 (1.22)	5.0	6 (4.6)	77 (59.2)
18	Skipped or stopped taking medicine because it made you feel bad?	4.33 (1.11)	5.0	5 (3.8)	84 (64.6)
19	Skipped, stopped, not refilled, or taken less medicine because of the cost?	4.45 (1.16)	5.0	6 (4.6)	100 (76.9)
20	Not had medicine with me when it was time to take it.	4.41 (1.15)	5.0	5 (3.8)	97 (74.6)

*The Arabic version of the ASK-20 questionnaire was utilized in this study.

Table 3 illustrates the validity outcomes of the ASK-20 tool, showing distinct variations between groups categorized as “Good-1” and “Poor-1.” Participants in the “Good-1” category demonstrated a lower mean score of 70.4 (SD 8.65) for ASK-20, indicating better adherence and understanding than a higher mean of 80.2 (SD 7.73) in the “Poor-1” group. Similarly, the TBC mean was notably lower in the “Good-1” group (11.5, SD 2.83) than in the “Poor-1” group (14.9, SD 2.61), reinforcing the disparity between the groups. These results reflect a significant association between the validity measures and adherence behaviors, with the “Good-1” group showing more favorable outcomes, as confirmed by narrower confidence intervals. This comparison underscores the reliability of these criteria in differentiating adherence levels among participants.

**TABLE 3:** Summary of validity outcomes.

Validity criteria	ASK-20 mean (SD)	ASK-20 95% CI	TBC mean (SD)	TBC 95% CI	Sample size (*n*)
Good-1	70.4 (8.65)	68.70–72.14	11.5 (2.83)	10.93–12.06	97
Poor-1	80.2 (7.73)	77.54–82.82	14.9 (2.61)	14.05–15.83	33

## Discussion

### Main findings

This study represents the first evaluation of the usability of the Arabic version of the ASK-20 questionnaire among Arab adults, addressing a critical gap in culturally adapted tools for assessing medication adherence in Arabic-speaking populations. The findings demonstrated that the Arabic version of the ASK-20 is a reliable and valid instrument with an internal consistency reliability (Cronbach’s alpha = 0.751) that meets acceptable standards. This aligns closely with the reliability observed in other language adaptations, such as Japanese and English. The results highlight several significant barriers to medication adherence, including cost-related issues and the unavailability of medication, which exhibited high-ceiling effects. Conversely, some items showed modest floor effects, such as those related to the inconvenience of taking medications multiple times a day. The study also identified notable differences in adherence behaviors between groups categorized by adherence levels. Participants in the “Good-1” adherence group displayed significantly lower mean ASK-20 and Total Barrier Count (TBC) scores compared to those in the “Poor-1” group, underscoring the tool’s ability to differentiate adherence levels effectively. These findings suggest that the Arabic ASK-20 can be a valuable tool for healthcare providers to systematically evaluate adherence behaviors and develop targeted interventions for Arab patients.

### Main discussion

This study’s results align with previous research findings and provide insights into the potential reasons behind the observed patterns. For instance, the high ceiling effects related to cost and medication availability may reflect the broader socioeconomic challenges faced by many Arab populations (16, 17). In many cases, out-of-pocket medical expenses can be substantial, and healthcare systems in some Arab countries may lack robust insurance coverage or access to affordable medication (18). The observed prominence of cost and medication availability as adherence barriers may be better understood in the context of the healthcare systems in Arab countries. Many nations in the region, including Egypt, Jordan, and parts of the Gulf Cooperation Council (GCC), face challenges related to fragmented insurance systems, out-of-pocket payments, and disparities in medication access. For example, in several lower- and middle-income Arab countries, public health services may be underfunded, and national health insurance schemes do not consistently cover all medications, especially for chronic diseases (16, 19). Even in wealthier countries such as Saudi Arabia, expatriate populations often rely on employer-based private insurance, which can vary widely in coverage (20). This mirrors findings from the Japanese validation study by Atsuta et al. (10), which also identified cost as a significant barrier (10). However, in Arabic, cultural norms around family financial responsibilities might exacerbate the impact of cost-related barriers, as patients may prioritize household expenses over personal health needs (21). The modest floor effects observed for questions about the inconvenience of taking medications multiple times a day suggest that this issue may be less problematic for the studied population. This could be due to the predominance of younger participants in the sample, who may have fewer difficulties managing complex medication regimens than older populations (22). Additionally, in cultures where family support is integral, patients might receive reminders or assistance with their medication schedules, mitigating this barrier (23). The differences in adherence behaviors between the “Good-1” and “Poor-1” groups further highlight the importance of systemic and patient-level factors. Participants with better adherence likely benefited from more precise communication with healthcare providers and more substantial personal commitment to health management (24). The Arabic ASK-20’s ability to capture these distinctions underscores its utility as an evaluative tool, particularly in contexts where healthcare literacy and patient engagement vary widely. The slightly lower internal consistency reliability (Cronbach’s alpha = 0.751) compared to the original English version [Cronbach’s alpha = 0.85, Hahn et al. (8)] may be attributable to linguistic and cultural differences in how specific questions are interpreted (8). For example, culturally specific attitudes toward medication use and trust in healthcare providers could influence responses (25). Furthermore, variations in healthcare system structures between Arab countries and Western contexts might impact the perceived relevance of specific questions (26). Comparisons with other studies, such as the derivation and validation of the ASK-12 tool by Matza et al. (9), reveal consistent themes in adherence research. Both tools highlight the central role of patient-provider communication and the influence of health beliefs on adherence behaviors (9). Overall, the study’s findings contribute to the global validation of the ASK-20 tool while emphasizing the need for context-specific adaptations. Healthcare providers can design more effective interventions to improve adherence by addressing financial, systemic, and cultural barriers. ASK-20 total score represents the cumulative response value across all 20 items on the 5-point Likert scale, capturing the degree to which participants experience adherence barriers, with higher scores indicating greater barriers. In contrast, the Total Barrier Count (TBC) is a simplified metric that counts the number of items for which the participant selects a response indicating the presence of a barrier (typically responses rated 4 or 5 on the scale). While the total score reflects the intensity and frequency of barriers, the TBC reflects the number of distinct types of barriers the individual encounters. Both measures offer complementary insights: the total score indicates severity, whereas the TBC identifies the breadth of adherence challenges.

### Strengths and limitations

One of the primary strengths of this study is its rigorous translation and validation process, which ensured that the Arabic ASK-20 questionnaire was both linguistically accurate and culturally relevant. Semantic equivalence with the original English version was rigorously maintained by implementing a systematic methodology encompassing forward translation, expert review, back-translation, and pilot testing. This ensures the tool is appropriate for Arabic-speaking populations and provides reliable insights into adherence barriers. Additionally, the study’s inclusion of a non-disease-specific population enhances the generalizability of the findings, allowing the Arabic ASK-20 to be applied across diverse patient groups. The robust statistical analyses, including examining floor and ceiling effects, further strengthen the validity of the results. Regarding the limitations, the cross-sectional design precludes the assessment of changes in adherence behaviors over time or the impact of interventions. Another limitation is the potential underrepresentation of adherence barriers unique to Arab cultures, such as social stigma or healthcare accessibility challenges, which the existing ASK-20 framework may not fully capture. Another limitation is the age distribution of the sample, with nearly half (49.2%) of participants aged 18–24 years. This younger demographic may include individuals who do not prescribe medications, which could influence how they perceive or report adherence barriers. Although the ASK-20 was administered in a general context rather than condition-specific, limited real-life medication use in this subgroup may have resulted in underreporting or less nuanced responses to certain barrier items. Although the ASK-20 was culturally adapted for Arabic speakers, its fixed item structure may limit its ability to capture adherence barriers unique to Arab populations, such as social stigma, reliance on traditional healing practices, or regional healthcare access disparities. These culturally specific factors may not be adequately reflected in the standardized items, potentially leading to underestimating their impact. Convenience sampling via social media platforms may have introduced selection bias, limiting participation to individuals with internet access and higher digital literacy. As a result, the findings may not fully represent the broader Arab adult population, particularly those in rural areas or with limited access to technology. The sample consisted predominantly of male participants (69.2%), which may affect the generalizability of the findings to female patients. Women may experience unique adherence barriers influenced by sociocultural roles, health beliefs, or healthcare access patterns that were not fully captured in this study.

## Conclusion

This study represents the first evaluation of the Arabic version of the ASK-20 questionnaire, providing a culturally adapted and validated tool for assessing medication adherence barriers among Arabic-speaking populations. The findings underscore the reliability and utility of the ASK-20 in identifying critical adherence barriers, particularly those related to cost and medication availability. These results contribute to the global validation of the ASK-20 tool, demonstrating its applicability across diverse cultural contexts. Future research should explore longitudinal validation of the Arabic ASK-20 to assess its stability over time and examine its applicability in specific clinical populations and healthcare settings across the Arab world.

## Data Availability

The raw data supporting the conclusions of this article will be made available by the author, without undue reservation.
